# Hypothermia and neonatal morbimortality in very low birth weight preterm infants

**DOI:** 10.1590/1984-0462/2022/40/2020349

**Published:** 2021-10-04

**Authors:** Rafaelle Cristine Oliveira Cordeiro, Daniela Marques de Lima Mota Ferreira, Heloísio dos Reis, Vivian Mara Gonçalves de Oliveira Azevedo, Airan dos Santos Protázio, Vânia Olivetti Steffen Abdallah

**Affiliations:** a Universidade Federal de Uberlândia, Uberlândia, MG, Brazil.; b Instituto Federal de Educação, Ciência e Tecnologia da Bahia, Irecê, BA, Brazil.

**Keywords:** Hypothermia, Infant, very low birth weight, Indicators of morbidity and mortality, Quality of health care, Hipotermia, Recém-nascido de muito baixo peso, Indicadores de morbimortalidade, Qualidade da assistência à saúde

## Abstract

**Objective:**

To assess the prevalence of hypothermia in the delivery room, at admission, and 2 to 3 hours after admission in the neonatal intensive care unit (NICU), factors associated and possible relationship with morbidity and mortality in preterm infants with very low birth weight (VLBW).

**Methods:**

Cross-sectional study with data collection based on a retrospective review of medical records and including infants born in 2016 and 2017, with birth weights <1500g, and gestational ages <34 weeks. Data about VLBW preterm infants, maternal data and temperature in the delivery room were analyzed. Hypothermia was considered when axillary temperature <36°C. For statistical analysis, the chi-square test or G test, canonical and Spearman correlation, and logistic regression were used.

**Results:**

149 newborns (NB) were included in the study. The prevalence of hypothermia in delivery room, at admission to the NICU and 2 to 3 hours after admission was 25.8%, 41.5% and 40.2%, respectively. The temperature of NBs was directly proportional to gestational age (p<0.010), birth weight (p<0.010), and Apgar score (p<0.050). There was an inverse association with hypothermia in the delivery room and cesarean delivery (OR 0.25; p=0.016).

**Conclusions:**

Hypothermia was a prevalent problem in the studied population. The neonatal temperature was directly proportional to gestational age, birth weight and Apgar score. Hypothermia was associated with maternal factors, such as cesarean delivery. It is necessary to implement and improve strategies for its prevention.

## RESUMO

**Objetivo:**

Avaliar a prevalência da hipotermia na sala de parto, à admissão e 2 a 3 horas após a admissão na Unidade de Terapia Intensiva Neonatal (UTIN), fatores associados e possível associação com morbimortalidade em recém-nascidos pré-termo de muito baixo peso (RNPT MBP).

**Métodos:**

Estudo transversal, com coleta retrospectiva de dados em prontuários dos RNPT MBP nascidos em 2016 e 2017, com peso <1500g e idade gestacional <34 semanas. Foram analisados dados dos RNPT MBP, da mãe e a temperatura na sala de parto. Foi considerada hipotermia a temperatura axilar <36°C. Para análise estatística, foram utilizados teste do qui-quadrado ou teste G, correlacçã canônica e de Spearman, e regressaã logística.

**Resultados:**

Fizeram parte do estudo 149 recém-nascidos (RN). A prevalência da hipotermia na sala de parto, à admissão na UTIN e 2 a 3 horas após a admissão, foi de 25,8, 41,5 e 40,2%, respectivamente. A temperatura do RN foi diretamente proporcional à idade gestacional (p<0,010), ao peso ao nascimento (p<0,010) e ao índice de Apgar (p<0,050). Observou-se associação inversa entre hipotermia na sala de parto e parto cesáreo (OR 0,25; p=0,016).

**Conclusões:**

A hipotermia foi um problema prevalente na população estudada. A temperatura do RN foi diretamente proporcional à idade gestacional, peso ao nascimento e Boletim de Apgar. Esteve também associada a fatores maternos, como parto cesáreo. Portanto, faz-se necessário a implementação e aprimoramento de estratégias para a sua prevenção.

## INTRODUCTION

Hypothermia is the result of an imbalance between heat loss and production.^1^ The World Health Organization (WHO) defines the range of 36.5 to 37.5 °C as normal axillary temperature for newborns (NB) and as hypothermia when below 36.5 °C.^1.2^

Very-low birth weight preterm newborns (VLBW PTNB), that is, those weighing less than 1500g, are more likely to drop body temperature after delivery. Therefore, one must adopt practices to minimize heat loss and provide heat. It is well established that measures such as maintaining adequate delivery room temperature, careful assessment of maternal temperature, use of radiant heat source, plastic bag and cap, respiratory support with heated and humidified gases, and incubator with adequate temperature control help prevent hypothermia in these patients.^3^

It is also known that hypothermia in newborns is associated with a range of morbidities, such as hypoglycemia, hypoxia, metabolic acidosis,^4^ peri-intraventricular hemorrhage (PIVH),^5^ necrotizing enterocolitis (NEC),^5^ late sepsis^6^ and bronchopulmonary dysplasia (BPD);^5^ as well as increased mortality,^7^ increasing the chance of death by 1.64 times when present at the admission of the NB to the Neonatal Intensive Care Unit (NICU).^8^ With all that in mind, WHO brings NB's thermal control among the ten proposed recommendations to address premature birth in order to reduce infant mortality.^9^ In addition, hypothermia prevention measures are among the pillars of care for newborns in the “Golden Hour”, a term applied in the context of care in the first hour of life to quickly stabilize patients, which has had a positive impact on the short- and long-term outcomes of neonates.^10^ ^-^ ^12^

However, keeping the NB warm after birth is a frequent problem in neonatology services worldwide.^1^ ^,^ ^2^ ^,^ ^6^ ^,^ ^8^ A study by Almeida et al.,^8^ from the Brazilian Network of Neonatal Research, reported hypothermia in 44% of NBs with gestational age between 23 and 33 weeks in the delivery room and 51% on admission to the NICU.

Given the importance of preventing hypothermia in the outcomes of VLBW PTNB and the need to know the reality to outline actions that guarantee the improvement of health care for this population, the objective of this study was to assess the prevalence of hypothermia after birth and in hours of admission to the NICU, as well as the associated maternal and neonatal factors, and the possible relationship with morbidity and mortality in VLBW PTNB from a public university hospital.

## METHOD

Cross-sectional, exploratory study with convenience sampling, in which data were obtained based on a retrospective review of medical records of newborns born between January 1, 2016 and December 31, 2017, in a public university hospital. The study was approved by the institution's Research Ethics Committee (Process No. 2,062,170, of May 14, 2017).

In this study, VLBW PTNB (<1500g) and with gestational age less than 34 weeks were included, and those weighing less than 500g, with congenital malformations or who died in the delivery room were excluded. Over the study period, 203 VLBW PTNB were born. Of these, 30 of them, whose birth weight was less than 500g, were excluded, as well as 15 who died in the delivery room and nine who had congenital malformations. Thus, 149 newborns were included in the sample.

In the medical records, two sets of variables were analyzed: related to newborns and related to their mothers. The neonatal variables analyzed were: weight (grams), gestational age (weeks), Apgar score in the 1st and 5th minutes of life, need for resuscitation — defined as the need for positive pressure ventilation in the delivery room—use of a plastic bag and double cap (plastic cap plus wool or cotton cap), NB temperature at five minutes of life in the delivery room, NB axillary temperature upon NICU admission and 2–3 hours after, and temperature normalization time for newborns who were admitted hypothermic.

Regarding the newborns’ outcomes, late sepsis (clinical and/or with microbiological evidence), presence of BPD (defined as use of oxygen at 28 days of life), PIVH, NEC (Bell's stage II and III, modified by Walsh and Kliegman),^13^ retinopathy of prematurity (ROP) and hospital death. The maternal variables assessed were: type of pregnancy (single or multiple), type of delivery (vaginal or cesarean), axillary temperature of the mother during delivery and presence of peripartum hemorrhage. The delivery room temperature was also analyzed. The mothers’ and newborns’ temperatures were measured in degrees Celsius (°C) and considered axillary temperature hypothermia (T) when <36°C (moderate and severe hypothermia) for the assessment of related factors and conditions. After admission, hypothermia was classified as mild (36.0 to 36.4°C), moderate (32.0 to 35.9°C) and severe (<32.0°C).^2^

The difference in frequency of hypothermia in VLBW PTNB in the delivery room, at admission and 2 to 3 hours after admission to the NICU, was verified by the Yates correlation. The possible association between hypothermia and health problems in NBs was verified by the chi-square test and G test. A possible association between the temperature of the newborns (in the delivery room and at admission) and the temperature of the environment was verified by Spearman's correlation, while a possible relationship between the temperature of the newborns at three moments (in the delivery room, at admission and 2 to 3 hours after admission) and neonatal variables was verified by Canonical Correlation. The normality of the data in the different tests was verified by the Shapiro-Wilk and D’Agostino-Pearson tests.

To verify the association between hypothermia and the set of maternal and neonatal variables, we applied a logistic regression model. To do so, both sets of variables were treated separately. For the set of maternal variables, hypothermia was considered a dependent variable and two models were tested: initial (in which all predictor variables were considered) and final (in which only the most relevant variables were considered). For the selection of the most relevant variables, the Stepwise method was used, based on the Akaike Information Criterion (AIC) to choose the best model. For the set of neonatal variables, hypothermia was considered an independent variable and controlled by gestational age and the need of resuscitation. An additional test was also carried out to check the influence of resuscitation (independent variable) on the occurrence of hypothermia in newborns (dependent variable). In all logistic regression tests, the overall adjustment of the model was verified by calculating the Pseudo R2 index. The chance of occurrence of factors (Odds Ratio, OR) and its 95% confidence interval (95%CI) were also calculated.

The frequency comparison tests (chi-square test and G test), the correlations (Canonical and Spearman) and the normality analyses (Shapiro-Wilk and D’Agostino-Pearson) were performed in BioEstat 5.0.^14^ Logistic regression and the Stepwise method were performed in R-Studio,^15^ using the glm and step functions, respectively, and the “modEvA” and “mfx” packages. In all analyses, the level of significance adopted was 0.05.

## RESULTS

**Figure 1:**
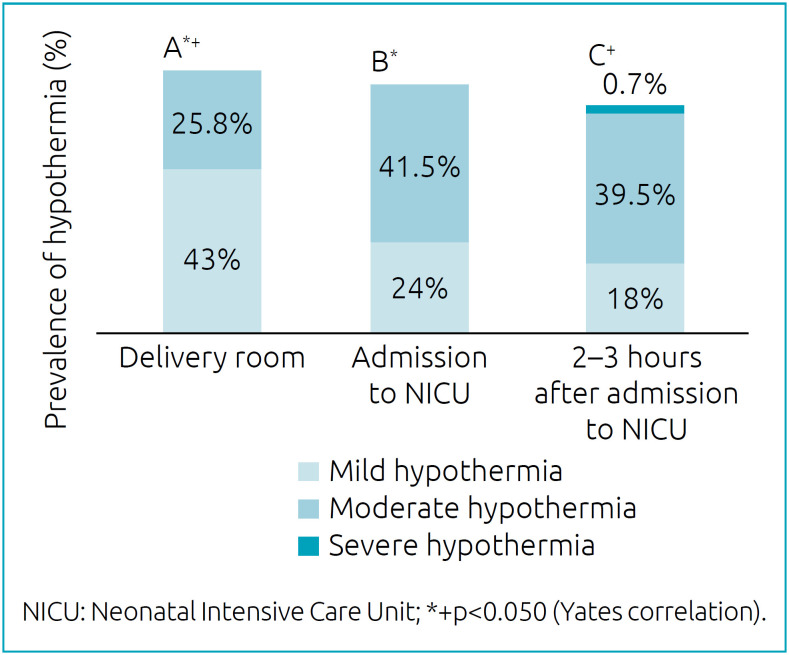
Prevalence of mild, moderate and severe hypothermia according to the moment in very low birth weight preterm newborns born in 2016 and 2017. Uberlândia, Minas Gerais.

The maternal characteristics and the 149 VLBW PTNB included in the study are listed in Table 1. The temperature was measured in 128 of the newborns with five minutes of life in the delivery room and in 147 upon admission to the NICU and then 2 to 3 hours later. Moderate hypothermia was observed, respectively, in 25.8 and 41.5% of newborns in the delivery room and on admission to the NICU. After 2 to 3 hours in the NICU, 39.5% of NBs still had moderate hypothermia and 0.7% had severe hypothermia (Figure 1). There was a significant increase in the occurrence of moderate and severe hypothermia in newborns at admission to NICU (p=0.032) and 2 to 3 hours after admission (p=0.043) when compared to five minutes of life in the delivery room. The newborns who were admitted to the NICU with hypothermia needed, on average, 7 hours (±5 hours) to normalize the temperature. Only one RN did not use a double cap and plastic bag. All newborns were transported in a heated incubator.

**Table 1:** Maternal and neonatal characteristics. Uberlândia, Minas Gerais.

Maternal data (n=133)		
	Mother’ temperature (°C) – mean±SD	36.1±0.8
	Peripartum hemorrhage – n (%)	18 (13.5)
	Single pregnancy – n (%)	110 (82.7)
	Cesarean delivery – n (%)	96 (72.2)
Neonatal data (n=149)		
	Gestational age (weeks) – mean±SD	27.8±2.5
	Male – n (%)	74 (49.7)
	Weight (grams) – mean±SD	971±280
	1st minute Apgar <7 – n (%)	87 (58.4)
	5th minute Apgar <7 – n (%)	21 (14.1)

SD: standard deviation

The mean temperature in the delivery room was 24.6±1.6°C, with 57 (43.2%) births below 25°C and 21 (15.9%) below 23 °C. The temperature in the delivery room was directly proportional to the temperature of the NB upon admission to the NICU (p<0.036), but not to the NB's temperature in the delivery room itself (p=0.688). The environment temperature during delivery was measured in 116 mothers, with mean of 36.2±0.8 °C, of which 32 (27.6%) were hypothermic. The NB’ temperatures in the three different moments was directly proportional to birth weight, gestational age and 1st- and 5th-minutes Apgar (Table 2).

**Table 2:** Association between temperature of very low birth weight preterm newborns at three different momentos and gestational age, birth weight and 1st and 5th-minute Apgar score. Uberlândia, Minas Gerais, 2016 and 2017.

	NB's temperature in the delivery room			NB's temperature upon admission to NICU			NB temperature 2–3 hours after admission to NICU		
	T≥36°C	T<36°C	p-value	T≥36°C	T<36°C	p-value	T≥36°C	T<36°C	p-value
Gestational age (weeks)*	28.5±2.1	26.4±2.4	<0.010^a^	28.5±2.3	26.9±2.6	<0.010^a^	28.5±2.3	26.8±2.5	<0.010^a^
Birth weight (grams)	1,029±267	821±252	<0.010^a^	1,060±253	831±255	<0.010^a^	1,064± 251	838±267	<0.010^a^
1st minute Apgar*	5.7±2.3	4.3±2.7	<0.050^a^	5.6±2.4	4.5±2.7	<0.050^a^	5.9±2.4	4.3±2.6	<0.050^a^
5th minute Apgar*	8.4±1.3	7.5±1.6	<0.050^a^	8.3±1.4	7.5±1.9	>0.050^a^	8.4±1.2	7.4±1.9	<0.010^a^

NB: newborn; NICU: Neonatal Intensive Care Unit; T: temperature

a Canonical Correlation

* described as mean ± standard deviation.

Death occurred in 43 (28.9%) of the 149 NBs, reaching 44.3% of hypothermic NBs and 18.6% of non-hypothermic NBs on admission to the NICU (Table 3). In the univariate analysis, PIVH (p=0.042), late sepsis (p=0.004) and death (p=0.001) were associated with moderate and severe hypothermia upon admission to the NICU. However, the same was not observed in relation to BPD, NEC, ROP and the need for resuscitation in the delivery room (Table 3). When adjusting the logistic regression for gestational age and need for resuscitation, the variables did not show a statistically significant association with hypothermia on admission to the NICU (Table 4). Moderate and severe hypothermias on admission to the NICU were also not associated with the need for resuscitation in the logistic regression (OR 1.08, 95%CI 0.52–2.31; p=0.822).

**Table 3:** Association between moderate and severe hypothermia upon admission to the Neonatal Intensive Care Unit and morbidities and death in very low birth weight preterm newborns born in 2016 and 2017. Uberlândia, Minas Gerais.

	Hypothermic NB — T<36°C		Non-hypothermic NB — T≥36°C		p-value
	Total^a^	n (%)	Total^a^	n (%)	
Resuscitation at birth	61	45 (73.8)	86	62 (72.1)	0.854^b^
PIVH	57	32 (56.1)	85	33 (38.8)	0.042^b^
Late sepsis	57	34 (59.7)	82	29 (35.4)	0.004^b^
Necrotizing enterocolitis	60	3 (5.0)	88	9 (10.2)	0.239^c^
BPD	36	23 (63.9)	74	35 (47.3)	0.102^b^
ROP	33	10 (30.3)	69	21 (30.4)	0.989^b^
Death	61	27 (44.3)	86	16 (18.6)	<0.001^b^

Analysis not adjusted to the sample size. PIVH: peri-intraventricular hemorrhage; BPD: bronchopulmonary dysplasia; ROP: retinopathy of prematurity;

a total newborns analyzed for each variable;

b chi-square test;

c test G.

**Table 4:** Logistic regression for the presence of hypothermia (T<36 °C) in very low birth weight preterm newborns in the delivery room and upon admission to the Neonatal Intensive Care Unit, according to maternal and neonatal variables. Uberlândia, Minas Gerais, 2016 and 2017.

		Odds Ratio	95%CI	p-value	Odds Ratio	95%CI	p-value
		Initial model containing all variables			Final model containing only the variables selected in the Stepwise method		
Hypothermia in VLBW PTNB in the delivery room							
Mothers’ and delivery^a^							
	Peripartum hemorrhage	0.53	0.11 a 2.05	0.392	–	–	–
	Cesarean delivery	0.29	0.09 a 0.92	0.034	**0.25**	**0.08 a 0.78**	0.016
	Multiple gestation	0.41	0.12 a 1.23	0.129	–	–	–
	Maternal hypothermia	2.71	0.99 a 7.57	0.054	**2.29**	**0.86 a 6.19**	0.096
Hypothermia in VLBW PTNB upon admission to the NICU							
Neonatal^b^							
	PIVH	1.58	0.73 a 3.42	0.234	–	–	–
	Late sepsis	1.78	0.84 a 3.79	0.126	–	–	–
	NEC	0.06	0.06 a 1.28	0.132	–	–	–
	BPD	2.42	0.74 a 8.77	0.153	–	–	–
	ROP	0.98	0.33 a 2.87	0.980	–	–	–
	Death	2.02	0.78 a 5.26	0.141	–	–	–

95%CI: 95% confidence interval; VLBW PTNB: very low birth weight preterm newborns; NICU: Neonatal Intensive Care Unit; PIVH: peri-intraventricular hemorrhage; NEC: necrotizing enterocolitis; BPD: bronchopulmonary dysplasia; ROP: retinopathy of prematurity;

a multiple logistic regression;

b logistic regression adjusted for gestational age and need for resuscitation.

As for maternal and gestational characteristics, assessed in a logistic regression model, cesarean delivery (OR 0.25, 95%CI 0.08–0.78; p=0.016) had an inverse relationship with the newborn's temperature <36 °C at delivery room, with no association between NB hypothermia in the delivery room and peripartum hemorrhage, multiple pregnancy and maternal hypothermia (Table 4).

## DISCUSSION

This study showed that hypothermia after birth and in the first hours after admission to the NICU was a frequent event in the population studied and that it was associated with maternal, neonatal factors and increased morbidity and mortality.

Despite efforts, hypothermia remains a frequent problem in neonatal care services worldwide, varying from 32 to 85% in prevalence.^16^ In this study, an increase in the frequency of moderate hypothermia in the VLBW PTNB was observed upon admission to the NICU and 2 to 3 hours later, in relation to the temperature at five minutes of life in the delivery room (Figure 1), the first being directly related to the temperature in the delivery room. Despite the finding that in only 15.9% of deliveries the room temperature was below the range recommended by the neonatal resuscitation guideline (23–26°C),^17.18^ in 43.2% of these cases, it was below 25°C. Studies have shown that the delivery room temperature ≥25°C is related to higher temperatures in newborns with gestational age <29 weeks.^19^ ^,^ ^20^

Thus, the temperature of the delivery room can justify the difference in the temperatures of the NBs between the first two moments evaluated, which is corroborated by the fact that the distance between the delivery room and the NICU, at the place of this study, is short, requiring a few minutes for transportation, which minimizes the influence of other factors. In addition, PTNBs have limited heat production capacity, and when the exposure to cold remains, the compensatory mechanisms of hypothermia are exhausted, leading to a drop in body temperature and its adverse effects.^16^ The average values of gestational age and birth weight of the studied population are characteristic of extreme PTNBs, as well as extreme low birth weight (Table 1).

In this study, as described in the literature, the newborn's temperature was directly proportional to gestational age and birth weight (Table 2), which was expected, since the characteristics of prematurity favor heat loss and reduce heat production.^7^ ^,^ ^8^ The same proportionality was found in relation to the Apgar Score (Table 2); however, there was no relationship with the need for resuscitation in the delivery room. Current evidence shows that newborns who have received more interventions in the delivery room (positive pressure ventilation, intubation, cardiac massage, medications) and, consequently, have shown a lower Apgar score, also have lower temperatures than those who did not require resuscitation. Laptook et al.^7^ found that the temperature of the NB at admission to NICU was 0.05 °C higher for each point of increase in the Apgar Score at 5 minutes.

Measures for preventing loss and providing heat, such as the use of a cap and a plastic bag, which are described in the literature as capable of raising the temperature of newborns with ≤28 weeks of gestational age to 0.5 °C,^19^ ^,^ ^21^ were used for most newborns. However, other resources, such as the heated gases during resuscitation and transportation, as well as a thermal mattress, were not available at the time of the study. The heating and humidification of gases can reduce the incidence of hypothermia by up to 35% and prevent its most severe degrees.^22^ Singh et al.^3^ found an increase from 27 to 46% in the proportion of newborns with gestational age <30 weeks admitted with adequate temperature with the use of a thermal mattress, but this has been associated with a significant increase in hyperthermia and is recommended only for newborns under 1000g.^3^ ^,^ ^12^

After 2–3 hours of admission to the NICU, 40.2% of the newborns had temperature <36 °C (Figure 1) and took, on average, 7 hours for normalization. This may result from the NB, upon arriving at the Intensive Care Unit (ICU), being subjected to various procedures, such as umbilical catheterization, surfactant administration and other medications that can negatively interfere with thermal control due to manipulation and opening of hatches in the incubator.^21^ Since these procedures are also essential to assit them during the “Golden Hour”, for respiratory and cardiovascular stabilization and nutritional support,10,11 actions must be coordinated, efficient and fast, carried out in a temperature-controlled environment.

In the univariate analysis (Table 3), PIVH, late sepsis and death were shown to be associated with the newborn's hypothermia upon admission to the NICU. However, after logistic regression analysis (Table 4), no association with the assessed outcomes was found. Although the relation between hypothermia and neonatal morbidity and mortality is well established, studies are divergent. Lyu et al.,^5^ when evaluating 9,833 newborns with gestational age less than 33 weeks, found lower rates of severe neurological injury, NEC, severe ROP and nosocomial infection when the admission temperatures ranged from 36.5 to 37.2 °C. Laptook et al.^7^ reported an inversely proportional relationship between newborns’ temperature at admission to the NICU and the incidence of late sepsis, but there was no relationship with PIVH, time of invasive mechanical ventilation and NEC; it was also shown that the temperature at admission is inversely related to in-hospital mortality, with a 28% increase in mortality for each 1 °C drop in body temperature.

In this study, among the maternal and gestational variables evaluated, there was no significant association between maternal and newborn hypothermia in the delivery room (Table 4). In addition, newborns born by cesarean delivery were less likely to develop hypothermia when compared to natural birth (Table 4). However, the literature describes that fetal temperature depends on maternal temperature and that, therefore, preventing maternal hypothermia contributes to the prevention of neonatal hypothermia.^23^ ^,^ ^24^ In addition, it has been shown that maternal temperature below 36 °C is associated with hypothermia in newborns at five minutes of life.^8^ Neuraxial anesthesia (spinal and epidural), generally used in cesarean deliveries, is associated with mild maternal hypothermia.^25^ Horn et al.^25^ found that newborns born to mothers undergoing warming up before and during elective cesarean section had significantly higher central temperatures and umbilical vein pH.

Although most studies highlight the risks of hypothermia in the delivery room and upon admission to the NICU, as well as the interventions carried out in order to prevent them, this study shows that the first hours in the NICU is also a critical moment, which reinforces the need to ensure adequate thermal control of this population during the whole period of hospitalization.

Several studies have reported a reduction in the occurrence of hypothermia through the implementation of bundles, which consist of adopting a set of simple measures to prevent it in the delivery room and during the transport of the newborn to the NICU, in addition to the continuing education of the team. Such measures resulted in an increase of up to 0.6°C in the average temperature of newborns in the delivery room and upon admission to the NICU, and a reduction in the percentage of individuals with temperature <36.0°C.^26^ ^-^ ^29^ This study is part of the initial stage of a project aimed at building a bundle for the prevention of hypothermia in VLBW PTNB. Based on the reality of the institution and on data obtained, strategies for the prevention of hypothermia were established and implemented.

Despite the importance of diagnosing hypothermia and establishing prevention strategies, this study had the following limitations: the cross-sectional retrospective design, with loss of data due to incomplete medical records; convenience sampling, without calculation of sampling power, which determined a small number of participants and could jeopardize the study of associations between variables; and the difficulty of generalizing the results. In addition, it does not allow determining whether there is a causal relationship between hypothermia and the outcomes in NBs, or whether this is only a marker of neonatal severity. These facts point to the importance of conducting new studies.

Moderate and severe hypothermia were a common problem in the population studied. The newborn's temperature was directly proportional to gestational age, birth weight and Apgar Score. It was also associated with maternal factors such as cesarean delivery. Therefore, a permanent process of implementation and improvement of strategies for its prevention is needed, including simple and accessible measures such as training and awareness of the team involved in care.
